# Recent trends in preparation and biomedical applications of iron oxide nanoparticles

**DOI:** 10.1186/s12951-023-02235-0

**Published:** 2024-01-08

**Authors:** Yu Qing Meng, Ya Nan Shi, Yong Ping Zhu, Yan Qing Liu, Li Wei Gu, Dan Dan Liu, Ang Ma, Fei Xia, Qiu Yan Guo, Cheng Chao Xu, Jun Zhe Zhang, Chong Qiu, Ji Gang Wang

**Affiliations:** 1 State Key Laboratory for Quality Ensurance and Sustainable Use of Dao-di Herbs, Artemisinin Research Center, and Institute of Chinese Materia Medica, China Academy of Chinese Medical Sciences, Beijing, 100700 China; 2 School of Pharmacy, Yantai University, No. 30, Qingquan Road, Laishan District, Yantai, Shandong China

**Keywords:** *Iron oxide nanoparticles*, *Synthesis*, *Applications*, *Nanomedicine*, *Nanocarrier*

## Abstract

The iron oxide nanoparticles (IONPs), possessing both magnetic behavior and semiconductor property, have been extensively used in multifunctional biomedical fields due to their biocompatible, biodegradable and low toxicity, such as anticancer, antibacterial, cell labelling activities. Nevertheless, there are few IONPs in clinical use at present. Some IONPs approved for clinical use have been withdrawn due to insufficient understanding of its biomedical applications. Therefore, a systematic summary of IONPs’ preparation and biomedical applications is crucial for the next step of entering clinical practice from experimental stage. This review summarized the existing research in the past decade on the biological interaction of IONPs with animal/cells models, and their clinical applications in human. This review aims to provide cutting-edge knowledge involved with IONPs’ biological effects in vivo and in vitro, and improve their smarter design and application in biomedical research and clinic trials.

**Graphical Abstract:**

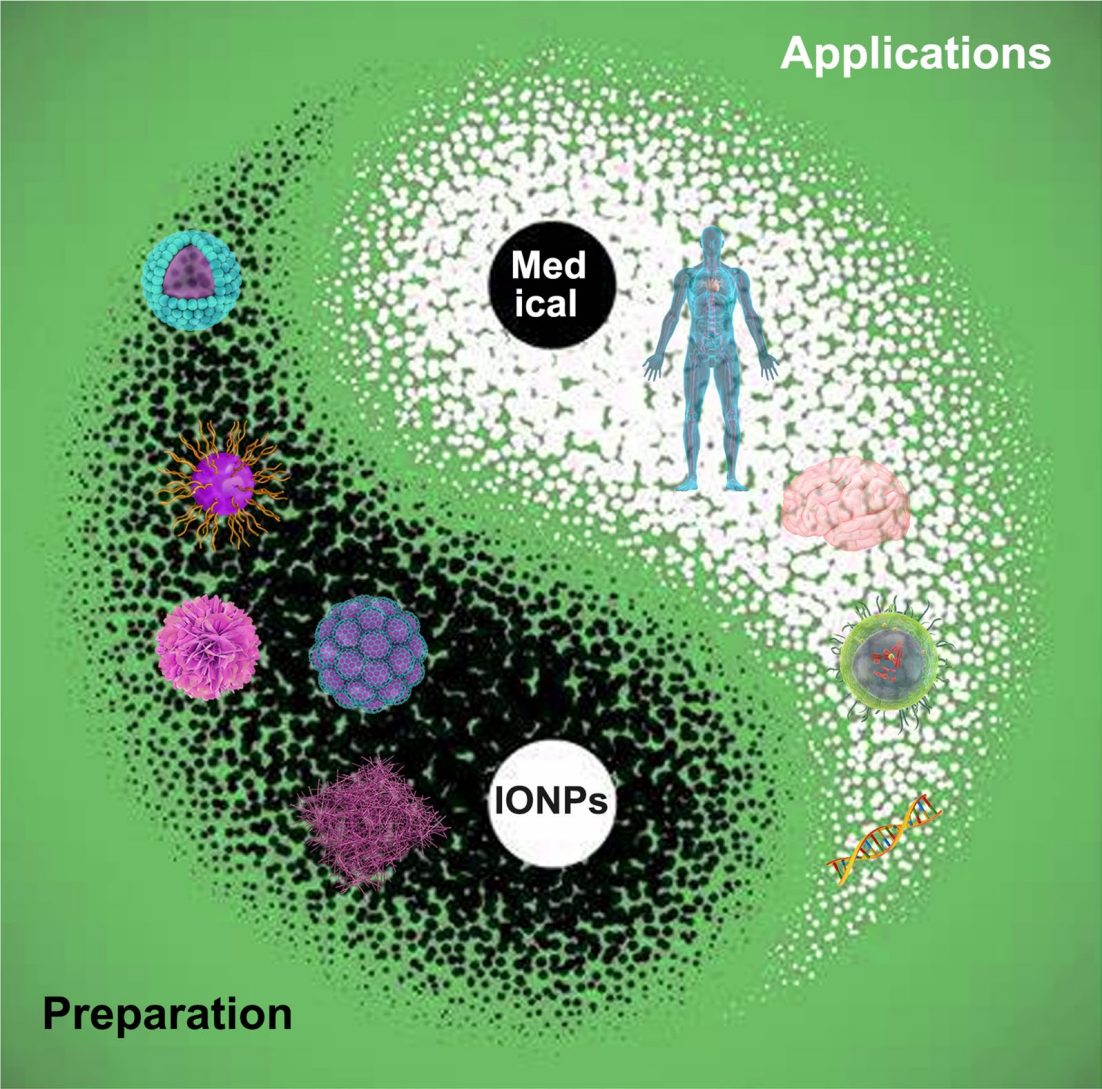

## Introduction

**Fig. 1:**
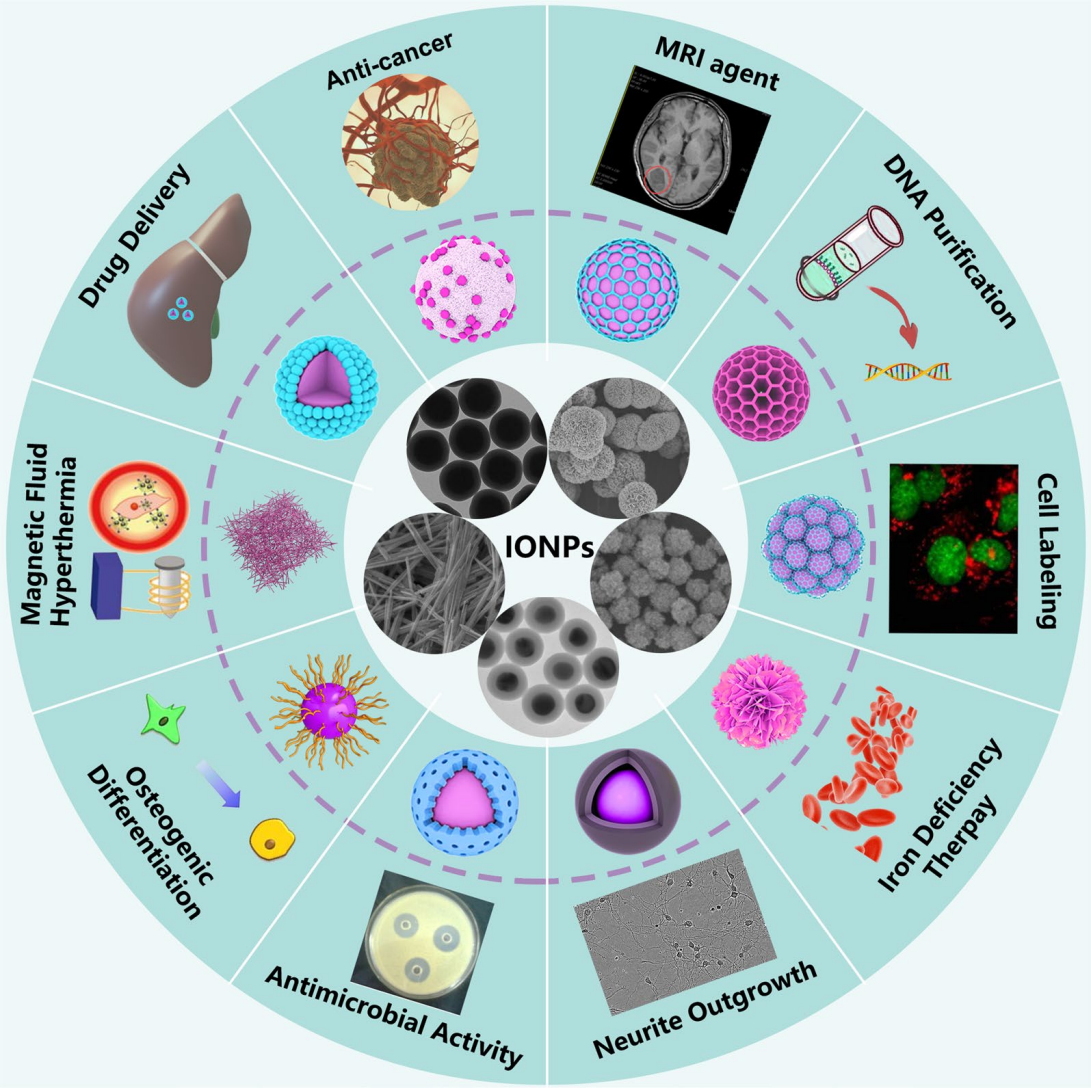
The biomedical applications of iron oxide nanoparticles. IONPs: iron oxide nanoparticles. MRI: magnetic resonance imaging

With the continuous expansion of the field of nanotechnology, the demand for nanoparticles in various industries is increasing. Nanomedicine is an important component of nanotechnology, which is mainly used for medical diagnostics and drugs delivery [1]. Magnetite (Fe_3_O_4_), hematite (*α*-Fe_2_O_3_), maghemite (*γ*-Fe_2_O_3_), and mixed ferrites are considered to be the main representative of iron oxide nanoparticles (IONPs) [2]. Due to their well-biocompatibility, fine biodegradability, low toxicity, and strong magnetism, IONPs have been widely employed in the biomedical fields, such as magnetic resonance imaging (MRI), targeted drug delivery, cancer immunotherapy and hyperthermia mediators (Fig. 1) [3–5]. Data have shown that superparamagnetic IONPs (SPIONs) can be used as potential drugs for the treatment of tumors. SPIONs can act as contrast agents for MRI, but also they can be used to carry out hyperthermia on cancer tissue under external magnetic field [6, 7]. Additionally, the modified SPIONs are assessed as platforms for delivering drugs or genes [8]. SIONPs also showed great antibacterial activity with minimum inhibitory concentration of about 100 ppm [9]. IONPs show a promising prospect in treating reactive oxygen species-related diseases, however they may pose a greater risk when exposed to human body. In order to design safe and effective IONPs for biomedical applications, there is an urgent need to summarize the preparation and biomedical applications of IONPs in different animal models, cell types or in the clinic [10].

**Fig. 2:**
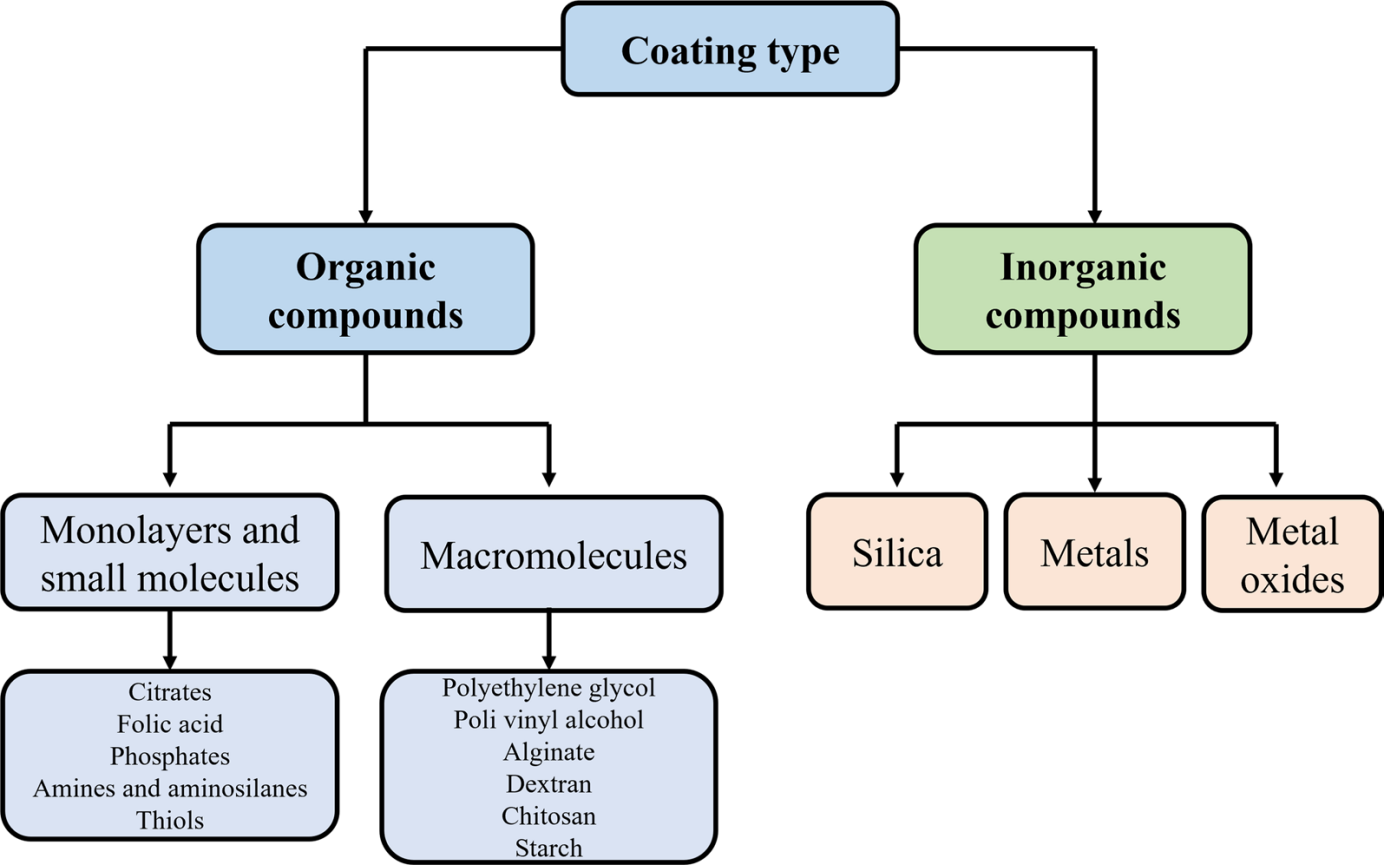
Different coating type on the surface of iron oxide nanoparticles

Several IONPs are the firstly approved type of metal–organic NPs for clinically or preclinical trials by the European Medicines Agency and United States Food and Drug Administration, such as Resovist®, Feridex®, and Feraheme®. However, a number of initially approved IONPs-based MRI contrast agents are withdrawn because of their severe failure in clinical trials. Surprisingly, recent study have indicated that IONPs with size smaller than 5 nm are promising MRI contrast agents [11]. With the gradual in-depth knowledge of the IONPs, the biocompatibility and toxicity of IONPs are primary determined by their size, while their surface coating molecules and functional group profoundly influence the bio-interaction between IONPs and biological system [12, 13]. It was reported that the coating material and thickness impact on the degradation rate and prothrombotic activity of IONPs [14]. The results showed that carboxymethyl dextran coated IONPs degraded faster in simulated body fluid than those coated with silica, and showed the least prothrombotic properties. In addition, the thickness was inversely proportional to the degradation rate. Besides, studies have demonstrated that the same IONP might show different biocompatibility or toxicity in different cell type or humans, which is also the predominant reason to hinder the application of IONPs in biomedical field [15, 16]. Hence, it is necessary not only to summarize the size, surface coatings and functional groups of IONPs (Fig. 2), but also to summarize the biomedical applications of IONPs in different animal models, cell types and humans, so as to promote the comprehensive understanding of IONPs by researchers and provide guidance for accelerating the clinical application of IONPs-based nanomedicine.

This review aims to provide a comprehensive overview of recent progress on the synthesis of IONPs, the biological interaction in different animal models and cell type, as well as the clinical application of IONPs, focusing on researches published from 2013 to the present. In the first part of this review, the most frequently used preparation techniques are summarized due to their low cost and high reproducibility. Then, we focus on the animal models’ studies of IONPs, including biocompatibility, bio-distribution, metabolism, bio-clearance. Secondly, we detailed describe the latest in vitro studies in tumor or non-tumor cells. Finally, clinical studies in human are introduced. This review may provide novel and more comprehensive understanding of IONPs ranging from synthetic methods, applications in different animal models, tumor and non-tumor cell lines, to their clinical applications, and further promote their development of biomedical applications.

## Synthesis of iron oxide nanoparticles

**Fig. 3:**
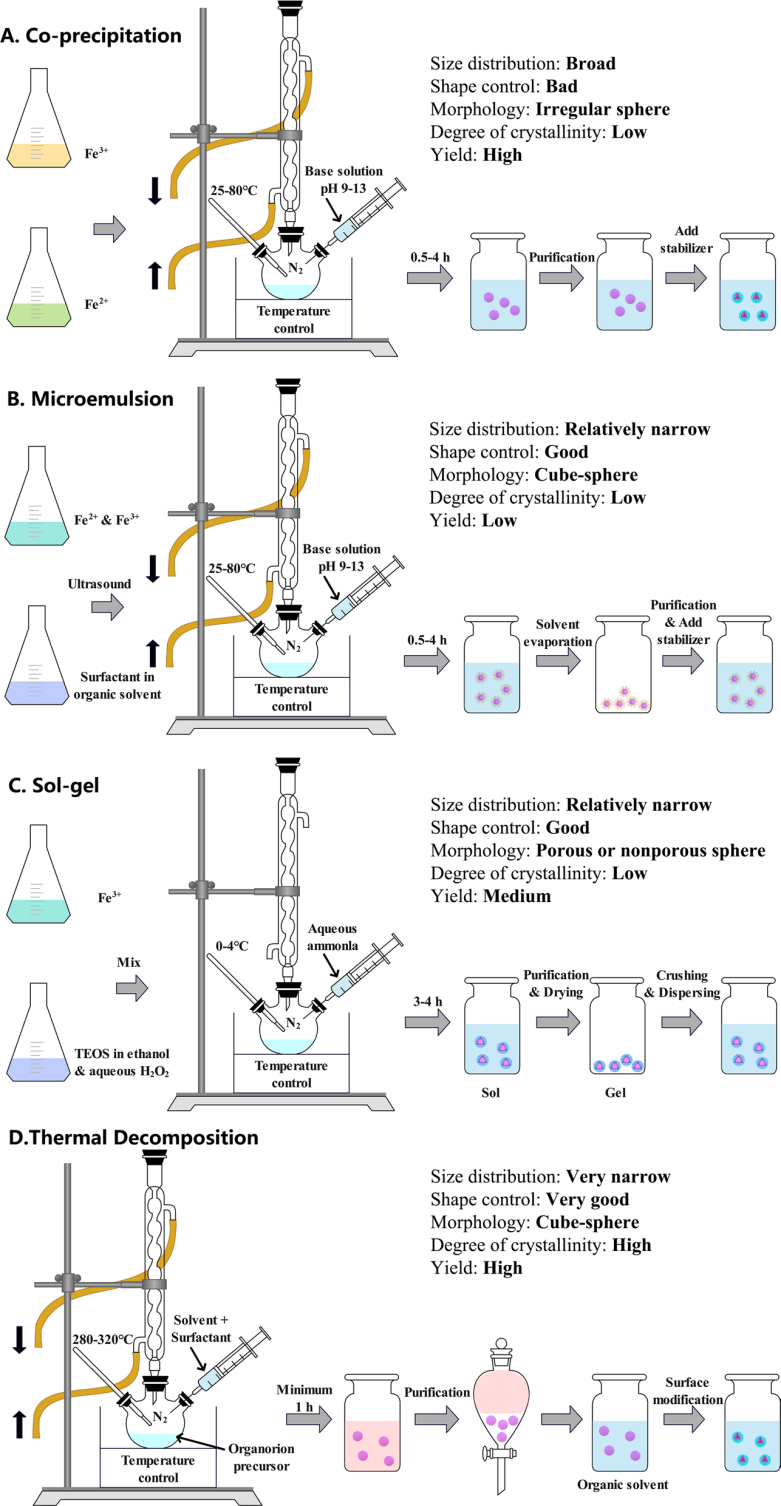
Schematic illustration of strategies to prepare iron oxide nanoparticles

Common synthetic methods for IONPs include three main categories: chemical methods, physical methods, biological synthesis methods. 90% of IONPs are synthesized via chemical methods, and the remaining 10% is obtained by physical or biosynthetic approaches [17, 18]. Chemical methods (Fig. 3) mainly include co-precipitation, micro-emulsion, sol–gel, and thermal decomposition, which the most efficient route for IONPs [19]. Representational physical methods are powder ball milling, electron beam lithography, aerosol, and gas phase deposition. Although the yield of physical methods is high, only 10% IONPs can actually be used for application because of the complexity in changing the target particle size and structure [20]. At present, IONPs are rarely synthesized via physical methods. The biological synthesis methods are primary completed through microbial enzymes or plant phytochemicals, which belongs to green chemistry [21]. IONPs synthesized through green synthesis show higher biocompatibility when compared to commercial IONPs [22–24]. The products obtained from biosynthetic methods are low yield with wide size distribution.

Co-precipitation method is the most efficient and effective chemical synthesis approach with broad size distribution and high yield. However, the products of co-precipitation method are poor size distribution, low crystallinity and large polydispersity [25]. The chemical reaction for co-precipitation method is as follows: Fe^2+^  + 2Fe^3+^  + 8OH^−^ → Fe_3_O_4_ + 4H_2_O, Fe_3_O_4_ + 2H^+^  → γFe_2_O_3_ + Fe^2+^  + H_2_O [26]. Micro-emulsions are composed of two incompatible liquids: oil-in-water and water-in-oil. The main strength of micro-emulsion method is that the size, nucleation and agglomeration of IONPs can be controlled. However, the crystallinity and yield of IONPs are relatively low. Additionally, the residual surfactants may influence the property of IONPs [27]. The schematic view of the micro-emulsion method is provided [28]. Sol–gel method is widely adopted strategy to synthesize silicon modified IONPs. The most significant advantage of sol–gel method is low cost, and the synthesis of IONPs with porous or nonporous sphere. The urgently need to be solved of this method is the by-product residue, which requires further purification before IONPs can be applied [29]. Schematic of different stages of sol–gel process can be found [30]. The size, shape, and dispersion of IONPs synthesized in thermal decomposition are under superior control, but the crystallinity of the products is low. Most seriously, the thermal decomposition synthesis route is not eco-friendly with longer synthesis time [31]. Patsula described how to synthesize IONPs by thermal decomposition method in detail [32].

## Applications of IONPs in animal models

IONPs have been widely exploited in various animal models and cell types, but the bio-distribution, bio-clearance, biocompatibility and toxicity of IONPs in different studies have shown significant differences. The main reason for this phenomenon is that the physical parameters of IONPs used in different studies are high variability. The shape, size and surface properties are the primary factors that determine the properties of IONPs and affect the biological interaction between IONPs and biological system [33]. The shapes of IONPs mainly include rod, spherical, cube and worm. Shape mainly affects bio-distribution, bio-clearance, and biocompatibility. The short rod IONPs mainly gather in the liver, while the long rod IONPs dominantly accumulate in the spleen. In addition, short rod IONPs are quickly cleared from the body through urine or feces, while long rod IONPs possess a longer blood circulation time [34]. Size primary influences the uptake rate, half-life time, distribution, and excretion of IONPs. IONPs with size smaller than 10 nm are rapidly uptake by liver and cleared by the kidney, while those large than 40 nm are mainly accumulated in spleen, which might contribute to better therapeutic efficacy or long-term toxicity issue [35]. Surface properties mainly include surface charge and modification. Surface charge plays the key factor to determine the dispersion stability and the distribution of IONPs in vivo. The surface modification or coating reduces the toxicity and improves the biocompatibility of IONPs compared to bare ones, especially when modified with hydrophilic polymers such as polyethylene glycol, hydroxyl or amino functional groups [36].

It is precisely due to the magnetic conductivity of IONPs that they can be widely used in biomedical fields such as bio-assays, magnetic drug targeting, tumor hyperthermia, nuclear magnetic resonance imaging and sensors. When iron atoms form a crystal, the arrangement of individual atoms will produce three different magnetic states: ferromagnetism (Fe_3_O_4_), ferrimagnetism (γFe_2_O_3_) and anti-ferromagnetism (αFe_2_O_3_) [37]. The IONPs commonly used in the biomedical field are mainly composed of Fe_3_O_4_ or γFe_2_O_3_, possessing magnetic targeting property to achieve directional delivery. In addition, anti-ferromagnetic IONPs are rarely investigated in the biological study, and are commonly studied in the field of optoelectronics, such as magnetic electron and spintronic devices [38]. It is worth noting that other anti-ferromagnetic metal materials (such as lanthanide base nitride) have been studied in the biomedical field as implants, such as hip and knee endo-prostheses and dental implants [39].

**Fig. 4:**
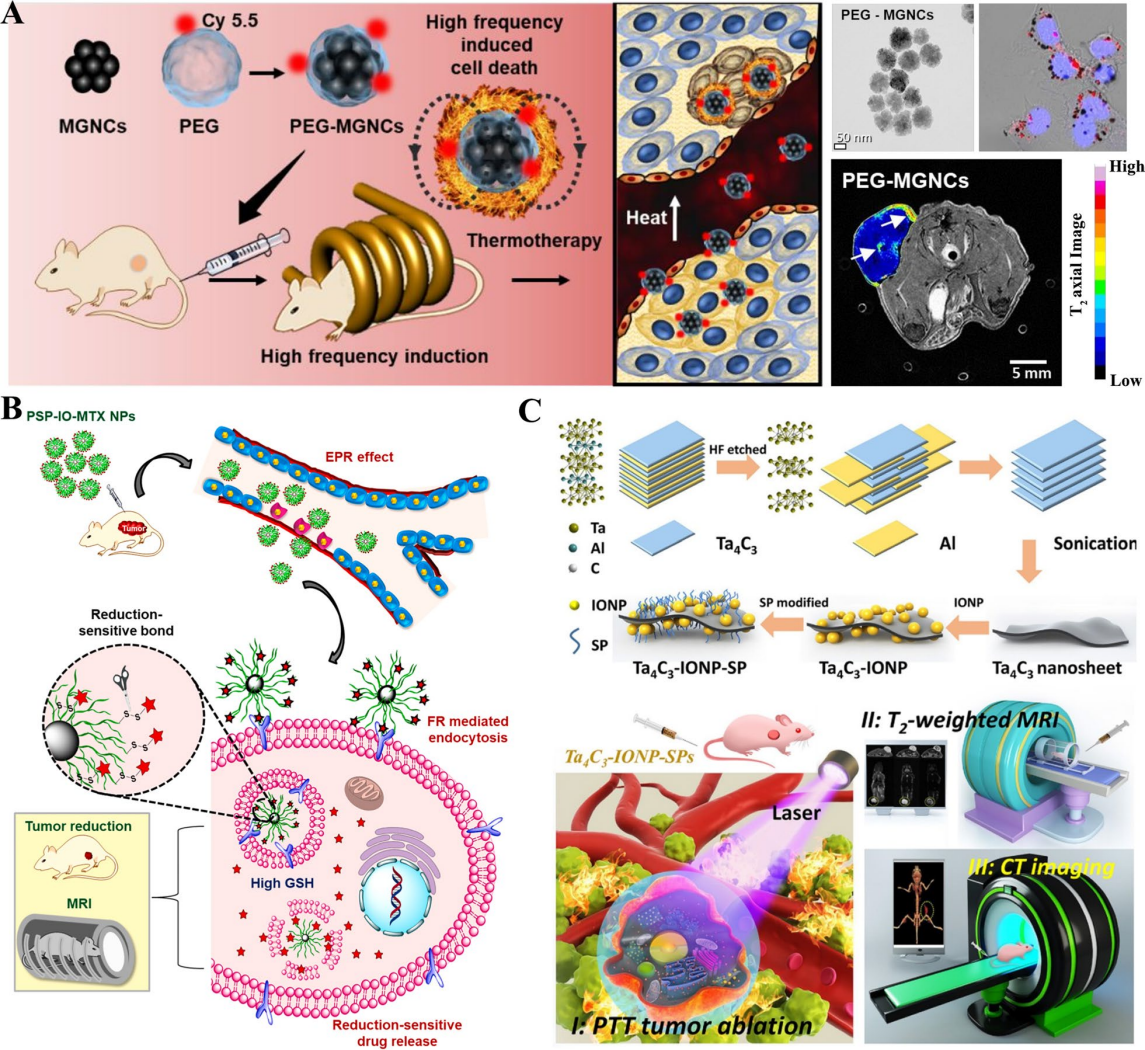
Schematic representation of synergistic anticancer effect under external magnetic field (**A**, [6]), magnetic resonance imaging (**B**, [49]), photothermal therapy (**C**, [85]) of different types of iron oxide nanoparticles. IONPs: iron oxide nanoparticles. MRI: magnetic resonance imaging. PTT: photothermal therapy. CT imaging: computed tomography imaging

The biocompatibility, bio-distribution, metabolism, and bio-clearance of IONPs in different animal models were summarized in this part. Importantly, an applied external magnetic field, MR imaging and photothermal therapy display a synergistic effect (Fig. 4). Iron oxide Sarah NPs (SaNPs, 22 mg/kg Fe) showed no adverse effects on healthy swine with or without alternating magnetic field (AMF). In addition, SaNPs was mainly distributed in the lungs, liver and spleen. Clearance of SaNPs showed a dose and time-dependent manner, which was predominantly eliminated through feces. Importantly, the SaNPs selectively accumulated in tumor tissue and regulated temperature by themselves when exposed to AMF [40]. Safety and biocompatibility of Maghemite/poly (d, l-lactide-co-glycolide)/chitosan NPs (γ-Fe_2_O_3_/PLGA/CS) were assessed in BALB/c mice. The results showed that γ-Fe_2_O_3_/PLGA was rapidly uptake by liver and spleen within 30 min, while the uptake of γ-Fe_2_O_3_/PLGA/CS in the liver was much less than γ-Fe_2_O_3_/PLGA. γ-Fe_2_O_3_/PLGA/CS did not gather in the spleen, which was in accord with the MRI results [41]. c (RGDyK) and D-glucosamine-grafted nanoprobe (Fe_3_O_4_@RGD@GLU) mainly accumulated in the liver and spleen in BALB/c mice. Magnetic targeting contributed to the accumulation of Fe_3_O_4_@RGD@GLU in the breast tumor region. Additionally, thermotherapy relatively increased the temperature in the tumor region, then inhibited tumor growth [42]. The impact of initial surface coating on magnetic iron and gold was investigated in mice after intravenous injection. The iron and gold were principally uptake in liver and spleen. Additionally, amphiphilic polymer-coated NHs could prolong the degradation when compared with polyethylene glycol-NHs. Fe_3_O_4_@macrophage membrane (Fe_3_O_4_@MM) could significantly reduce the tumor size in BALB/c nude mice over time after intravenous injection with Fe_3_O_4_@MM (2.5 mg/kg Fe). Fe_3_O_4_@MM could basically ablate the tumor with the aid of photothermal therapy [43]. Nine types of FeOx NPs (3–22 nm) were synthesized with multiple size and coating. The bio-distribution and clearance were investigated in mice, which indicated that the NPs (size at 3 nm and 11 nm) were rapidly distributed in liver and spleen, and excreted via urinary system [44]. Gogoi et al. [45] developed a Fe_3_O_4_ and La_0.75_Sr_0.25_MnO_3_ NPs for hyperthermia and chemotherapy. Single and double dose treatment of Fe_3_O_4_ and La_0.75_Sr_0.25_MnO_3_ NPs via intratumoral injection could significantly reduce 2.5 folds and 3.6 folds of fibrasarcoma tumor without any leaching or drainage observed in mice. Fe_2_O_3_@bovine serum albumin (Fe_2_O_3_@BSA) (0.15 mM/kg Fe) were mainly distributed in the liver, spleen, and kidney of rats after tail vein injection for 24 h, then cleared by kidney at 48 h without inducing any damage and side effects [46]. Additionally, zebrafish, as an emerging model to investigate the potential toxicity, has been used successfully to assess the potential risks induced by the IONPs. The result showed that carbon-modified α-Fe_2_O_3_ significantly reduced oxidative stress and apoptosis, which had higher biocompatibility than Fe_3_O_4_ [47, 48]_._

Poly (ethylene glycol)-l-arginine@IONPs (PEG-Arg@IONPs) were mainly uptake by liver, besides spleen, heart and kidneys in BALB/c model within 2 h. After 24 h, the PEG-Arg@IONPs were nearly excreted via kidney [49]. The difference of biocompatibility and biodistribution of IONs coated with citrate (citrate@IONPs, 2.4 mg Fe) were assessed in elderly and young healthy mice. The result indicated that there was an age-dependent effects on citrate@IONPs, which was reasonably biocompatible for young mice, while the liver and immune functions were slightly decreased in elderly mice. Spleen, liver and lungs were the main organ for iron biodistribution in young mice. For elderly mice, liver and kidneys were the predominantly accumulation organs [16]. The bio-distribution of IONPs@citrate, IONPs@curcumin, IONPs@chitosan, and ferrous sulfate were investigated in rats after gavage of 4 mg/kg IONPs for 10 days. The result showed that IONPs mainly accumulated in the liver. IONPs@chitosan or ferrous sulfate was accumulated in the spleen or kidney, respectively. IONPs@curcumin and IONPs@chitosan were mild toxic when compared with IONPs@citrate and ferrous sulfate [50]. IONPs-chloride, IONPs-lactate, and IONPs-nitrate (100 mg/kg) showed no obvious signs of toxicity in rats after oral administration for 14 days. Compared to IONPs@lactate and IONPs@nitrate, IONPs@chloride was the safest compound and induced less oxidative stress in rats [51]. IONPs functionalized with or without human albumin were both biocompatible in rats, which did not change the system hemodynamic or microcirculation. The size and surface coating influenced the accumulation time in organ. Pure IONPs and IONPs@human albumin were firstly gathered in liver, then in spleen and kidney during 24 h. Human albumin increased the circulation time of IONPs in rats [52]. After intravenous injection with 1.5 mg/kg of DMSA-IONPs, the DMSA-IONPs mainly gathered in spleen, liver and lung, then gradually graded into small size NPs over 90 days without inducing any toxicity in C57BL/6 mice [53]. Shen et al. [54] compared the MRI efficiencies of exceedingly small IONPs (ES-IONPs) in different size (below 5 nm), and found 3.6 nm was the best size. Moreover, a drug delivery system based on 3.6 nm ES-IONPs was built to enhance tumor target ability, the result indicated that the accumulation of ES-IONPs in tumor was higher than those in liver and spleen, which could be utilized as MR contrast agent. IONPs@polyethylene glycol multi-granule (PEG-MGNCs) were mainly accumulated in lung, and rapidly cleared via kidney. PEG-MGNCs (8 mg/kg) could enhance the hyperthermia efficacy in SCC7 tumor-bearing mouse model [12]. The cytotoxicity of PEG carboxyl-poly (ɛ-caprolactone) modified IONPs (PEG-PCCL-IONPs) mainly distributed in the spleen and liver after treated for 48 h in H22 tumor xenograft BALB/c mice, and remarkably decreased the tumor volume with good biocompatibility [55].

The biocompatibility of dextran-coated SPION (SPIONdex) was investigated in pig and mice model. The result indicated that SPIONdex (5 mg Fe/kg) was safe, and no complement activation-related pseudoallergy occurred after intravenous administration. Additionally, MRI indicated that liver signal intensity of SPIONdex could be detected after exposed for 24 h, which might be a candidate for MRI contrast agent [56]. Lactobionic acid (LBA) functionalized IONPs could enhance the release of ceftriaxone in albino rabbit model, and the plasma concentration of ceftriaxone was 14.46 ± 2.5 μg/mL, which was much higher than that in the control group [57]. The biological impact of SPION@PEG-COOH and SPION@PEG-NH_2_ was assessed in mouse, which revealed there was no difference in mouse after intrapulmonary administration. The SPIONs mainly accumulated in the lung and transient gathered in the liver. In addition, the SPIONs in the lung were gradually cleared with time, and returned to control value at 7 days [58]. The biocompatibility of PEG-coated SPIONs was investigated in Kunming mice. SPIONs@PEG mainly accumulated in the liver, spleen, and intestine, and gradually excreted via the hepatobiliary mechanism after 14 days [59]. The biocompatibility of SPION functionalized with tocopheryl-polyetheleneglycol-succinate (TPGS) or didodecyl-dimethyl-ammonium-bromide (DMAB) was investigated in mice. SPION-DMAB mainly accumulated in brain and spleen, while SPION-TPGS internalized in liver and kidney on the 7th days after gavage with 12.5 μg/kg SPION. On the 21st day, the oxidative stress was significantly reduced with Fe clearance [60]. SPIONs coated with l-cysteine could increase the adipose tissue in the inferior layer of the epidermis of mice after treated with 0.1 mg/kg SPIONs coated with l-cysteine for 7 days. Additionally, the concentration of iron remained unchanged in the spleen and blood after injection because the SPIONs were completely target to magnet region [61]. Poly (lactide)@SPIONs nanofibers were prepared and implanted in rats via peritoneal cavity for 6 months. Long-term MRI and histological analyses revealed that the degradation of this SPIONs nanofibers was quite slowly, as evidence that they were easily detected after 6 months post-implantation [62]. Bi-layer, which was consisted of oleic acid and methoxy-polyethylene glycol-phospholipid was coated in SPIONs, was named as SPION-PEG2000. The in vivo result showed that SPION-PEG2000 (12.5 mg/kg) induced necrosis in liver and kidney and inflammatory infiltration in lung [63]. The silica-coated SPION fluorescent NPs were mainly accumulated in kidney, liver, and lung, and did not cause obviously acute and chronic toxicity in mice [64]. SPION could enhance the formation of chondrogenesis in rats via activating the TGF-/SMAD signaling pathways [65]. Galactomannan (PSP001) functioned SPIONs could increase the accumulation of methotrexate (MTX) in the tumor site and decrease the toxicity of MTX in BALB/c mice, which provided an option for MRI imaging and targeted tumor therapy [66].

Ultra-small SPIONs (USPIONs) whose size below 5 nm were highly toxic with the lethal dosage at 100 mg/kg in the mice. However, USPIONs (size at 9.3 nm) showed no significantly toxicity and predominantly uptake in heart, liver, spleen, and lung. Meanwhile, different-sized of SiO_2_ and gold functioned USPIONs were synthesized, which revealed good biocompatibility in mice. The result indicated that the toxicity was related to the size of USPIONs [67]. A hepatocellular carcinoma targeted probe was developed by glypican-3 (GPC3)-specific aptamer (AP613-1) and USPIO (Apt-USPIO). The Apt-USPIO (1 mg Fe/mL) was excellent biocompatible in Kunming mice without damaging any vital organs. Importantly, Apt-USPIO could obviously target GPC3 on hepatocellular carcinoma in xenograft mice [68]. To sum up, majority of IONPs are non-toxic, and have well biocompatibility to the vital organs of studied animals (Table 1). Additionally, the toxicity of the IONPs mainly depends on the surface modification and coating.

**Table 1:** Summary of different types of iron oxide nanoparticles (IONPs) in animal models

Coating molecule	Name	Model	Dose	Days	Outcome	References
Polyethylene glycol	PEG-MGNCs	SCC7 tumor-bearing mouse	8 mg/kg	8 days	Enhance the hyperthermia efficacy	[12]
Citrate	Citrate@IONPs	Elderly and young healthy mice	2.4 mg Fe/kg	28 days	Reasonably biocompatible for young mice	[16]
Polyethylene glycol	SaNPs	Swine	22 mg IO/kg, 3.6 mg IO/kg	90 days	No adverse effects	[40]
Chitosan	γ-Fe_2_O_3_/PLGA/CS NPs, γ-Fe_2_O_3_/PLGA NPs	BALB/c mice	5 mg/kg	1, 24 h	No toxicity in vital organs	[41]
c(RGDyK)and D-glucosamine	Fe_3_O_4_@RGD@GLU	BALB/c mice	30 mg Fe/kg	8 days	Tumors on mice were obviously inhibited	[42]
Macrophage membranes	Fe_3_O_4_@MM NPs	BALB/c mice	2.5 mg Fe/kg	16 days	Significantly reduce the tumor size	[43]
Dopamine sulfonate, zwitterionic dopamine sulfonate, coryneine chloride	FeOx NPs	CD1 mice	1 or 4 mg Fe/kg		Rapidly distributed in liver and spleen, and excreted via urinary system	[44]
/	Fe_3_O_4_ NPs	Swiss mice	1, 2 mg Fe/kg	12, 22 days	Significantly reduce the tumor growth	[45]
6 − 7 bovine serum albumin	Fe_2_O_3_@BSA	SD rats	0.15 mM Fe/kg	24, 48 h	Efficiently cleared within 48 h	[46]
Poly (ethylene glycol)-L-arginine	PEG-Arg@IONPs	BALB/c mice	20 mg Fe/kg	24 h	Mainly uptake by liver, besides spleen, heart and kidneys	[49]
Citrate, curcumin, chitosan	IONPs@citrate, IONPs@curcumin, IONPs@chitosan	Wistar rats	4 mg Fe/kg	10 days	IONPs@curcumin and IONPs@chitosan were mild toxic	[50]
Chloride, lactate, nitrate	IONPs@chloride, IONPs@lactate, and IONPs@nitrate	Wistar rats	100 mg/kg	14 days	No signs of toxicity	[51]
Human albumin	IONPs@human albumin	Wistar rats	2 mg Fe/kg	24 h	Firstly gathered in liver, then in spleen and kidney	[52]
Dimercaptosuccinic acid	IONPs@DMSA	C57BL/6 mice	15 mg Fe/kg	7, 30, 60, 90 days	No toxicity	[53]
/	ES-IONPs	U-87 MG tumor-bearing nude mice	5 mg Fe/kg	28 days	Accumulate in tumor	[54]
Poly (ethylene glycol) carboxyl-poly(ɛ-caprolactone)	PEG-PCCL-IONPs	H22 tumor xenograft BALB/c mice	20 mg/kg	48 h	Mainly distributed in the spleen and liver	[55]
Dextran	SPIONdex	Pig model	5 mg Fe/kg	30 min	No complement activation-related pseudoallergy observed	[56]
Lactobionic acid	MNP-LBA	Albino rabbit	25, 50 mg/kg	24 h	Enhance the release of ceftriaxone	[57]
Polyethylene glycol-COOH, Polyethylene glycol-NH_2_	SPION@PEG-COOH and SPION@PEG-NH_2_	BALB/c mice	0.8 mM Fe/kg	28 days	Mainly accumulated in the lung	[58]
Polyethylene glycol	PEG·SPIONs	Kunming mice	2.5 mg Fe/kg	14 days	Primarily in the liver, spleen, and intestine,	[59]
Didodecyl-dimethyl-ammonium-bromide, tocopheryl-polyetheleneglycol-succinate	SPION-DMAB, SPION-TPGS	Swiss albino mice	12.5 μg Fe/kg	7 days	SPION-DMAB mainly accumulated in brain and spleen, while SPION-TPGS internalized in liver and kidney	[60]
L-cysteine	Cys-Fe_3_O_4_ NPs	BALB/c mice	0.1 mg Fe/kg	7 days	Increase the adipose tissue in the inferior layer of the epidermis of mice	[61]
Poly(lactide)	PLA@SPIONs	Sprague Dawley rats	2 cm^2^	6 months	Slow degradation	[62]
Oleic acid and methoxy-polyethylene glycol-phospholipid	SPION-PEG2000	Swiss albino mice	12.5–50 mg/kg	14 days	Induced necrosis in liver and kidney and inflammatory infiltration in lung	[63]
Silica	sub-5 SIO-Fl	CD-1 mice	10 mg/kg	7 weeks	No obviously acute and chronic toxicity	[64]
/	SPION	Sprague Dawley rats	10–40 μg/mL	8 weeks	Enhanced the formation of chondrogenesis	[65]
Galactomannan	PSP-IO NPs	BALB/c mice	10–50 mg/kg	14 days	Increased the accumulation of methotrexate in the tumor site and decrease the toxicity of methotrexate	[66]
/	USPIONs	ICR mice	100 mg/kg	7 days	No significantly toxicity	[67]
Glypican-3-specific aptamer	Apt-USPIO	Kunming mice	1 mg Fe/mL	30 days	Excellent biocompatible	[68]

## In vitro applications of IONPs

### IONPs in tumor cells

**Fig. 5:**
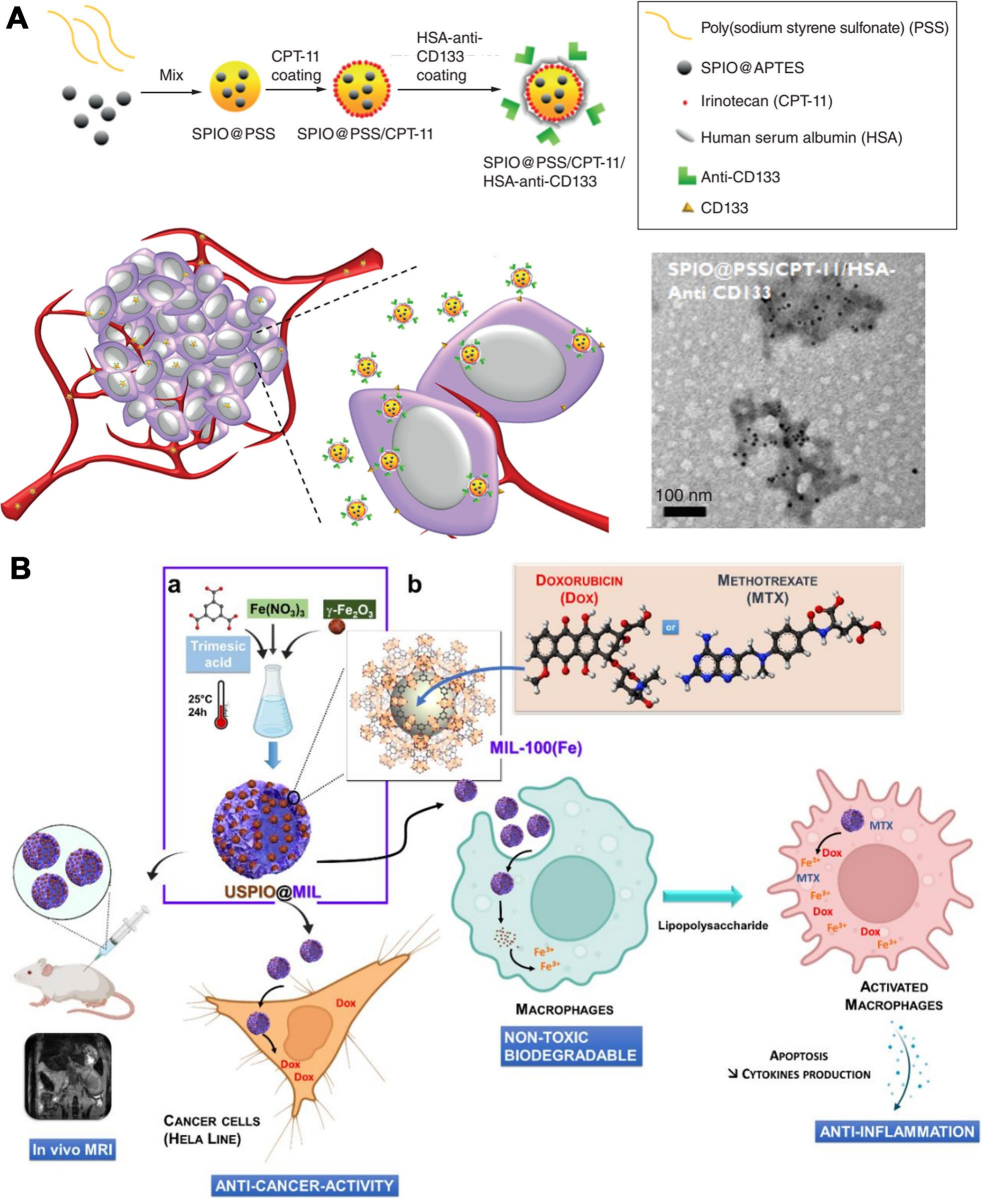
Schematic illustration of synthesis and applications of iron oxide nanoparticles in vitro. SPIO@PSS/CPT-11/HSA-anti-CD133 nanoparticles (**A**, [88]). USPIO@MIL-100(Fe) nano-objects (**B**, [109]). CPT-11: Irinotecan. HSA: Human serum albumin. PSS; Poly (sodium styrene sulfonate). SPIO: Superparamagnetic iron oxide

The IONPs target various type of tumor cells and induce tumor cell death without affecting normal cell viability (Table 2). The toxicity of IONPs in tumor cells is mainly related to the shape, surface modification, size, concentration and valence state. Importantly, an applied external magnetic field, radiofrequency generator irradiation, MR imaging and photothermal therapy display a synergistic anticancer effect (Fig. 5).

**Table 2:** Summary of different types of iron oxide nanoparticles (IONPs) in cell lines

Coating molecule	Name	Model	Dose	Days	Outcome	References
Poly (ethylenimine), poly(allylamine hydrochloride), poly(diallyldimethylammonium chloride)	IONPs-PEI, IONPs-PAH, IONPs-PDADMAC	A549 cell line	100 μg/mL	24 h	poly(allylamine hydrochloride) stabilized IONPs were the best biocompatibility	[69]
Polydopamine	Fe_3_O_4_@PDA	NK cell line	50 μg Fe/mL	12 h	It could regulate immune cells, inhibit tumor growth	[70]
Magnesium	Mg-γ-FeO	A549 cell line	0.1–250 mg Fe/mL	24 h	Significant cytotoxic effects	[71]
Polyethylenimine-calcium phosphate	SPIONs@PEI-CPs	A549 and HepG2 cell lines	10–60 μg Fe/mL	24 h	SPIONs@PEI-CPs were excellent biocompatibility, while SPIONs@PEI were remarkable cytotoxicity	[72]
Polyethylene glycol	IONPs	A549 cell line	0–250 μg Fe/mL		No significantly toxicity	[73]
Anti-αvβ6 antibodies	αvβ6-MIONPs	VB6 and H357 cell lines	0.2 mg Fe/mL	24 and 48 h	αvβ6- magnetic NP could enhance the killing potential of OSCC when combined with magnetic field	[74]
Chitosan	CS@IONPs	HSC-2 cell line	0.08–2.5 mg Fe/mL	48 h	No synergism with anticancer drugs; not completely rescue the X-ray-induced cell damage	[75]
Folate-chitosan-docetaxel	SPIONs coated with folate-chitosan-docetaxel	L929, KB and PC3 cell lines	0.005–0.08 μM	48 h	targeted cytotoxicity in cancer cells	[76]
Chitosan, growth factor domain, somatomedin B domain	IONPs/C, IONPs/C/GFD, IONPs/C/SMB	SKOV3 cell line	0.25, 0.5, 1 μg Fe/mL	24, 48 h	GFD + SMB showed synergistic effect	[77]
Cobalt and manganese	CoMn-IONP	ES-2 cell line	0–1000 μg Fe/mL	24 h	High saturation magnetization and heating efficiency	[78]
/	SPIONs-Serum	SKOV3 cell line	50–200 μg Fe/mL	24 h	Significantly inhibited the cell proliferation	[79]
Single-chain antibody, β-cyclodextrin, docetaxel	Fe_3_O_4_-scFv-β-CD- TXT	SKOV3 cell line	2 mg/mL	72 h	Continuously inhibited the growth of Skov3 ovarian cancer cells	[80]
Chitosan	Cs-coated SPIONs	HEK-293 cell line	100–500 μg Fe/mL	24, 48, 72 h	Non-toxic	[81]
/	γ-Fe_2_O_3_ NPs	Caco-2, HT-29, and SW-480 cell lines	0–500 μg Fe/mL	24 h	Carbohydrate and polymer coated on the surface of NPs enhanced the biocompatibility	[82]
Polyethylene glycol	Fe_3_O_4_@PEG	COLO-205 cell line	0–60 μg Fe/mL	24 h	Cytotoxicity to cancer cells	[83]
Silica	Fe@FeOx@SiO_2_ NPs	HCT116 cell line	100 μg Fe/mL	72 h	No cytotoxicity	[84]
Silica	Sub-5 nm silica@IONPs	Caco-2 cell line	10, 50, 100 μg/mL	24 h	Well biocompatible	[85]
Carboxylate, amine	IONPs	C10 cell line	5–200 μg Fe/mL	24 h	Cytotoxicity and oxidative stress in a dose-dependent manner	[86]
Aptamer, Au	Aptamer-Au@SPIONs	HT-29, CHO and L929 cell lines	10–100 μg Fe /mL	24 h	Concentration influenced the cytotoxicity	[87]
Poly (sodium styrene sulfonate)/irinotecan/human serum albumin-anti-CD133	SPIONs@PSS/HAS-anti-CD133	Caco2, HCT116, DLD1 cell lines	1–10 mg/mL	24 h	Inhibited the tumor cell viability in a dose-dependent manner	[88]
Dextran	University of Luebeck-Dextran coated SPION	Head and neck squamous cancer cell line	0.2–1.8 mM Fe	120 h	Decreased cell proliferation	[89]
Hyaluronic acid, HA-PEG10	HA-PEG10@SPIONs	SCC7 cell line	0.1–100 μg/mL	2 h	Remarkably decreased SCC7 cell viability	[90]
Dextran, hyaluronic acid, cisplatin	SEON^DEX−HA*CPt^	PC-3 cell line	10, 30, 50 μg Fe/mL	24 h	SPIONs with cisplatin induced apoptosis and necrosis	[91]
J591	IONPs	LNCaP, PC3, DU145, 22RV1 cell lines	48 h	48 h	No effect on cell viability	[92]
Poly(N-isopropylacrylamide-acrylamide-allylamine)	R11-PIONPs	PC3 and LNCaP cell lines	50–500 μg/mL	6, 24 h	Inhibited the tumor cell viability in a dose-dependent manner	[93]
Docetaxel	Fe_3_O_4_ NPs	DU145, PC-3, and LNCaP cell lines	1–100 μg/mL	72 h	Slightly cytotoxicity	[94]
Luteinizing hormone-releasing hormone receptor peptide and urokinase-type plasminogen activator receptor peptide	LHRH-AE105-IONPs	PC-3 cell line	10–100 ng/mL	24 h	Remarkably decreased PC-3 cell viability	[95]
Hyaluronic acid	FeO@HA NPs	L929 normal cell and MDA-MB-231 cancer cell	12.5–200 μg/mL	24, 48 h	High targeting specificity to cancer cells	[96]
/	Exceedingly small IONPs	MCF7 and 4T1 cell lines	0.8 mM Fe	24 h	Non-cytotoxicity	[98]
/	IONPs	4T1 cell line	100 μg Fe/mL	24 h	Decreased 4T1 cell viability to 48.5%	[99]
Arginine-methotrexate	Fe-Arg-MTX	MCF-7, 4T1, HFF-2 cell lines	50–800 nM	48, 72 h	Significantly decreased the cell viability	[100]
Macrophage membrane	FeO@MM	MCF-7 cell line	800 μg/mL	24 h	No toxicity	[43]
Dimercaptosuccinic acid	DMSA-SPION	MCF-7 cell line	50–500 μg/mL	0.5–72 h	Targeting breast cancer cells	[101]
Tantalum carbide	Ta_4_C_3_-IONP-SPs composite MXenes	4T1 cell line	12.5–200 ppm	24 h	Excellent biocompatibility	[102]
Poly(amidoamine) dendrimer-Pluronic P123/HSP90α	IPP/MB nanobeacon	MDA-MB-231 and MCF-10A cell lines	0.5–10 μg Fe/mL	48 h	Good cytocompatibility	[103]
Three bioengineered silks (MS1Fe1, MS1Fe2, and MS1Fe1Fe2)	H2.1MS1: MS1Fe1/IONPs	SKBR3 and MSU1.1 cell lines	0.19–25 μg/mL	72 h	Toxicity was observed when the concentration was more than 12.5 μg/mL	[104]
Silica	PVPMSFe	MCF-7, HFF2 cell lines	10–250 μg Fe	48, 72 h	No cell toxicity	[105]
Oleic acid, gelatin	IONPs coated with oleic acid-gelatin shell	HeLa cell line	2.5–25,000 ng/mL	48, 72 h	Higher therapeutic efficacy	[106]
Polycaprolactone	PCL-IONPs	HeLa cell line	10 μg doxorubicin	24 h	Cytotoxic effects on Hela cells	[107]
Protein conjugated glutaric acid	Pro-Glu-FeO	WI26VA, MCF-7 and HeLa cell lines	10–320 μg/mL	24 h	No toxicity in human normal lung cells, slight toxicity in MCF-7 and HeLa cells	[108]
Doxorubicin or methotrexate	USPIO(20)@MIL, USPIO(20)@MIL/MTX and USPIO(20)@MIL/Dox	Hela and RAW 264.7 cell lines	20, 50 μg/mL	12, 24 h	USPIO(20)@MIL showed low cytotoxicity to Hela cells, but no cytotoxicity to macrophages. USPIO(20)@MIL/MTX and USPIO(20)@MIL/Dox remarkably inhibited the cell viability in both cell lines	[109]
3-aminopropyl-triethoxysilane, aminodextran, and dimercaptosuccinic acid	IONPs-AD, IONPs-DMSA, IONPs-APS	HeLa cell line	0.05–0.5 mg Fe /mL	72 h	Low toxicity without morphological alteration	[110]
Heparin-Poloxamer	SPION@HP	HeLa cell line	0–500 μg/mL	48 h	Highly biocompatible	[111]
Poly(ethylene glycol)	Fe_3_O_4_@PEG	SGC7901/ADR cell line	0–20 μg/mL	48 h	EnhanceD cell apoptosis with low toxicity	[112]
Au, β-CD, SiO_2_	Fe_3_O_4_@Au@β-CD and Fe_3_O_4_@Au@SiO_2_ NPs	MGC-803 cell line	20, 50, 100, 200 μg/mL	24 h	Selectively uptaken by gastric cancer cells	[113]
Carboxymethyl cellulose, 5-fluorouracil	Fe_3_O_4_-CMC-5FU	SGC7901 cell line	0.05–1.0 μg/mL	24, 48, 72 h	Apparently antitumor effect	[114]
Atranorin	Atranorin@SPIONs	Gastric cancer stem cell line	1–100 μg/mL	24, 48, 72 h	Obviously inhibit gastric cancer stem cell proliferation	[115]
Poly (ethylene glycol)	γ-Fe_2_O_3_/CeO_2_@PEG	U87MG cell line	0.00045–2.7 mg/mL	24, 48 h	Induced cell death	[116]
Zinc	Zinc@SPIONs	U-87 MG cell line	1, 10, 25, 50, 100 μg/mL	12, 24 h	No cytotoxicity	[117]
Human serum albumin (paclitaxel)-Arg-Gly-Asp peptides	SPIOCs@HSA(PTX)-RGD	U-87 MG cell line	2–50 μg/mL	24 h	No cytotoxicity	[118]
Aurroshell gold	Aurroshell gold@hematite	U-87 MG cell line	5–1000 μg/mL	72 h	Remarkably killed glioblastoma cancer cell	[119]
Doxorubicin	Dox-IONPs	U251, bEnd.3 and MDCK-MDR1 cell lines	0.5–30 μg/mL	48 h	No cytotoxicity	[120]
Poly(acrylic acid), poly (serine ester), poly(ethylene glycol)	PICs	MC3T3-E1 and HepG2 cell lines	0.751 to 751 μM	24 h	Low cytotoxicity	[121]
Glutathione and cysteine	FePd IONPs	HepG2, AGS, SK-MEL-2, MG63, and NCI-H460 cell lines	5–20 μg/mL	1–7 days	Excellent biocompatibility	[122]
Silica	sIONPs	HuH7 cell line	0–160 sIONPs/cell	24,48 h	Excellent biocompatibility	[123]
/	USPIONs	PLC/PRF5 cell line	100 μg Fe/mL	48 h	Highly compatible	[124]
Pullulan	P-SPIONs	HepG2 and L-929 cell lines	25–100 μg/mL	24 h	Excellent biocompatibility	[125]
Zinc, cobalt	Zinc-IONPs, cobalt- IONPs	MG-63 and human bone marrow derived mesenchymal stem cell lines	10–500 μM	72 h	Short term acute cytotoxicity	[126]
Vascular endothelial growth factor, n-hydroxysuccinimide	IONPs@CD80 + VEGF	ATCCTM CRL-2836 cell line	0.1–100 μg/mL	24 h	Significantly reduce d aberrant cell proliferation	[127]
Hydroxyapatite,	IONPs@HA	MG-63 osteosarcoma cell line	20–120 μg/mL	48, 72 h	Marked toxicity	[128]
Chitosan, succinic anhydride, folic acid	IONPs@CS-FA/CS-SA	MG-63 osteosarcoma cell line	20 μM	72 h	Significantly inhibited cell proliferation	[129]
Hyperbranched polyester, dodecenyl succinic anhydride	FeO/HBPE-DDSA	OCI-LY3 cell line	0–100 mg/mL	24 h	No cytotoxicity	[130]
/	IONPs	diffuse large B-cell lymphoma cell line	0–1200 μg Fe/mL	48, 72 h	Remarkably inhibited the cell growth	[131]
Rituximab antibodies and Poly (ethylene glycol)	Fe_3_O_4_-PEG-nAb	Raji cell li ne	50 μg Fe/mL	72 h	Valence-dependent manner of Raji cell apoptosis	[132]
Methotrexate	FeO@MTX	Diffuse large B-cell lymphoma line	20–500 μg Fe/mL	24 h	Inducing cell apoptosis	[133]
/	IONPs-quantum dots	A20 mouse B-lymphoma cell line	5–100 μg/mL	12, 24, 48, 72 h	Regulate autophagy	[134]
Silibinin	IONPs@silibinin	A-498 cell line	0.001–10 μg/mL	96 h	Remarkably inhibited the cell growth	[135]
mAb G250	mAb G250-SPIONs	786–0 renal carcinoma cell line	10–100 μg/mL	12 h	No cytotoxicity	[136]
Gelatin, akermanite	Gel/Akr/Fe_3_O_4_/MWNT nanocomposite	G292 osteoblastic cells	0.125, 0.25, 0.50 mg/mL	24, 48, 72 h	Low cytotoxicity	[145]
Hydroxyapatite, collagen	FeHA/Coll	MG63 human osteoblast-like cell line	8.00 mm diameter and 3.00 mm high	72 h	Significantly promoted the cell proliferation	[146]
/	IONPs	Human primary adipose derived stem cell line	4–64 μg/mL	24 h	Affected the adipogenic and osteogenic differentiation	[147]
Antigen peptide	α-AP-fmNPs	BMDCs and dendritic cell 2.4 cell lines	0.3–48 μg/mL	24 h	No cell toxicity	[148]
/	SPIONs	Dendritic cell line	10, 25, 50 μg/mL	24 h	Nearly 100% of cells were labeled by the SPIONs	[149]
Citric acid, dextran	IONPs-CIT, IONPs-DEXT	THP1, NCTC 1469 cell lines	1.6–100 μg Fe/mL	24 h	No toxicity	[150]
/	SPIONs	Neurite	10 mM	48 h	Increased length and area of neurite	[151]
Glucosamine, poly(acrylic acid)	SPION-PAA, USPIO-PAA, USPIO-PAAGlcN	Mesenchymal stem cell line	100 μg/mL	24 h	Excellent biocompatibility	[152]
2,3-dimercaptosuccinic acid	γ-Fe_2_O_3_-DMSA	Human MSCs cell line	15–80 μg Fe/mL	2, 6, 24 h	No significant cytotoxicity	[153]
/	Ruicun	MSCs cell line	50–400 μg Fe/mL	24 h	Excellent biocompatibility	[154]
Curcumin	IONPs with curcumin	Bone marrow-derived mesenchymal stem cell line	0–1000 μg/mL	24 h	Dose-dependent cytocompatibility	[155]
Protein-specific molecularly imprinted polymers	MIPs	Human mesenchymal stem cell line	0.05, 0.1, 0.2 mM	24 h	High biocompatibility and low cytotoxicities	[156]
Citric acid	IONPs@CA	Endothelial cells and MC3T3-E1 cell lines	100 μg/mL	24, 48 h	Just affected cell viability	[157]
/	Magnetoferritin	Human MSCs cell line	0.01–3 μM	1 min	Biocompatibility	[158]
Silk fibroin	SPION@silk fibroin	Human bone marrow-derived MSCs cell line	2.5 mg Fe	21 days	Positively regulate the adhesion and proliferation	[159]
d-mannose	d-mannose (γ-Fe2O3)	Neural stem cell line	0.002–0.2 mg/mL	48 h	Slightly totoxicity	[160]

#### Lung carcinoma cells

The surface coating on IONPs plays a vital role in cellular uptake and biocompatibility. Rozhina investigated the cytotoxicity of three polycations-stabilized Fe_3_O_4_ NPs in lung carcinoma cells (A549 cells). The result indicated that poly (ethylenimine) (PEI), poly (allylamine hydrochloride) (PAH), and poly (diallyldimethylammonium chloride) (PDADMAC) did not change the magnetic property of IONPs. In addition, PAH coated Fe_3_O_4_ NPs were non-toxic and the most biocompatible, while the PEI showed the most toxic to A549 cells [69]. 50 μg/mL polydopamine coated with Fe_3_O_4_ were uptake by natural killer cells (NK) without changing their physiological properties, then NK cells could effectively kill A549 cancer cells with the help of an external magnetic field [70]. Mg-γ-FeO (0.25 mg/mL) decreased the cell viability of A549 cells with cell viability around 15% under an AMF (16.7 Ka/m, 110.1 kHz). However, the viability of A549 cells was not affected when the cells were treated with Mg-γ-FeO alone [71]. SPIONs@polyethylenimine-calcium phosphate (SPIONs@PEI-CPs) were designed to load doxorubicin and DNA, which was two kind of anticancer drugs. twofold of SPIONs@PEI-CPs were taken into A549 cells with the applied external MF, and remarkable inhibited the growth of A549 cells [72]. IONPs with core size of 11.3 ± 4.5 nm (below 250 μg Fe/mL) were internalized by A549 cells without causing any significant morphology changes, which suggested this IONPs was suitable for MRI contrast agents in vitro [73].

#### Oral squamous cell carcinoma cells

IONPs with αvβ6 antibodies were designed to target the oral squamous cell carcinoma (OSCC) tumor cells (VB6 cells). The synthetic IONPs could directly target αvβ6 overexpressing cells, and cause 85% cell death under AMF for 10 min [74]. The cytotoxicity of CS@IONPs was assessed in four types of human OSCC cell lines (Ca9-22, HSC-2, HSC-3, HSC-4) and three normal oral cell lines (HGF, HPLF, HPC). There was similar dose-dependent cytotoxic manner in OSCC and normal oral cell lines, which was biocompatible at low concentration (0.16–0.31 mg/mL), and cytotoxic at high concentration (1.25–40 mg/mL). Additionally, CS@IONPs showed synergism with 5-FU, abraxane and cisplatin in HSC-2 cells [75]. SPIONs coated with chitosan was used to delivery docetaxel, which was non-toxic to L929 cells at a concentration range of 100–1000 μg/mL. Docetaxel modified SPIONs showed dose-dependent toxicity on PC3 and KB cell lines whose 50% inhibitory concentrations (IC_50_) was 80 nM and 8.5 nM, respectively. KB cell lines, as a kind of oral cancer cells, was folate receptor positive, which contributed to the internalization of SPIONs [76].

#### Ovarian carcinoma

CS-coated IONPs had negligible cytotoxicity in SKOV3 cells after exposed for 24 and 48 h. However, fluorescein isothiocyanate-growth factor domain-somatomedin B domain functionalized IONPs obviously induced cell death (more than 40% cell died) under the concentration of 0.25 μg/mL [77]. Although the concentration of cobalt and manganese coated IONP nanoclusters (CoMn-IONP) increased to 300–1000 μg/mL, the cell viability only decreased 10–25% in ovarian cancer cells even with highly internalization efficiency. Additionally, CoMn-IONP nanoclusters (200 μg/mL) efficiently rose the temperature by 23 °C in ES-2 cells, while the IONP nanoclusters just elevated the temperature by 3 °C when exposed to AMF (26.9 kA/m, 420 kHz) [78]. SPIONs-Serum (50–200 μg Fe/mL) could significantly inhibited the cell proliferation in A2780 and SKOV3 cell lines for 24 h via inducing lipid peroxidation and ROS [79]. Fe_3_O_4_ functionalized with single-chain antibody (scFv), β-cyclodextrin (β-CD), and docetaxel (TXT) were designed for ovarian cancer therapy. The synthetic IONPs were excellent in normal cells, while stopped the growth of SKOV3 cells after 72 h treatment due to the sustained release of TXT [80].

#### Colorectal carcinoma

γ-Fe_2_O_3_/PLGA/CS (0.1–100 μg/mL Fe) were non-toxic to the HFF-1 cell line. Meanwhile, γ-Fe_2_O_3_/PLGA/CS remarkably decreased the T-84 cell viability to 61% under magnetic fluid hyperthermia [41]. The influence of ultrasonic irradiation on CS-SPIONs was assessed in HEK-293 cells. The CS-SPIONs were excellent biocompatible in any concentration within 72 h. 1.5 min sonication period with 67 W showed the best biocompatibility with 70% cells alive in 72 h [81]. The toxicity of various γ-Fe_2_O_3_ NPs was investigated in Caco-2, HT-29, and SW-480 cells. The result suggested that synthesis procedure and surface coating affected the uptake and toxicity of γ-Fe_2_O_3_ NPs in cancer cells, but not in normal cells. Carbohydrate and polymer coated on the surface of NPs enhanced the biocompatibility and internalization in epithelial colorectal adenocarcinoma cell lines [82]. The synthesized Fe_3_O_4_@PEGylation-TRAIL (0–60 μg/mL Fe) just showed cytotoxicity to cancer cells (COLO-205) but not to normal cells (HUVEC). Importantly, the Fe_3_O_4_@PEGylation-TRAIL indicated a slight phagocytosis by macrophage, which suggested its potential as a contrast agent for MRI [83]. Fe@FeOx@SiO_2_ NPs (100 μg/mL) showed no cytotoxicity to HCT116 cells even exposure for 72 h, while were high toxic in CCD112-CoN cells under the same incubation condition [84]. Sub-5 nm silica@IONPs were well biocompatible and non-toxic when treated Caco-2 cells with the highest concentration (100 μg/mL), which revealed their promising prospect in diagnosis and application [85]. Sharma, G compared the differences of cytotoxicity and the expression of redox-regulated gene in different surface modification IONPs in C10 cells. The result showed that carboxylated IONPs could induced cytotoxicity and oxidative stress in a dose-dependent manner, while the IONPs with amine surface modifications were non-cytotoxic to C10 cells [86]. Aptamer-Au@SPIONs at 10–100 μg/mL showed insignificant cytotoxicity in HT-29, CHO and L929 cell lines. Nevertheless, the cytotoxicity of aptamer-Au@SPIONs was positively correlated with concentration. In addition, aptamer-Au@SPIONs could induce the death of HT-29 cells when exposed to near infrared light (NIR) [87]. The cytotoxicity of SPIONs@poly(sodium styrene sulfonate)/irinotecan/human serum albumin-anti-CD133 (SPIONs@PSS/HAS-anti-CD133) were assessed in three kind of colorectal cancer cell line, including Caco2, HCT116, DLD1 cells. The result suggested that the SPIONs@PSS/HAS-anti-CD133 were highly biocompatible and inhibited the tumor cell viability in a dose-dependent manner. Furthermore. SPIONs@PSS/HAS-anti-CD133 exhibited highly cytotoxicity in Caco2, HCT116 cells with radiofrequency generator irradiation for 30 min [88] (Fig. 5A).

#### Head and neck squamous cell carcinoma

SPION functioned with university of Luebeck-dextran inhibited cell proliferation of head and neck squamous cancer cells UT-SCC-60A and UT- SCC-60B in a dose- and time-dependent manner without inducing oxidative stress and inflammatory responses [89]. 2 mg/mL hyaluronic acid (HA) and HA-PEG10 coated SPIONs (HA-PEG10@SPIONs) showed an excellent heating ability which could attain 42 °C in 600 s, which remarkably decreased SCC7 cell viability to 25% with hyperthermia. Nevertheless, the cell viability of NIH3T3 cell was comparable to the control under the same treatment. The difference was primary due to the selective uptake of HA-SPIONs to SCC7 cells [90]. 100 μg/mL PEG-MGNCs did not induce cytotoxicity in SCC7 cells without AMF, while obviously cytotoxicity was observed in SCC7 cells with cell viability to 42.9 ± 6.2% under AMF (19.5 kA/m, 389 kHz). However, IONPs@PEG did not induce cell death under the same condition [12].

#### Prostatic carcinoma

SPIONs with a dextran and HA were synthesized to delivery cisplatin (SEON^DEX−HA*CPt^). The biocompatibility of SEON^DEX−HA*CPt^ was investigated in PC-3 cells, which indicated that SPIONs with cisplatin induced apoptosis and necrosis under prolonged exposure in a dose-dependent manner [91]. The conjugation of IONPs to J591 antibody had no effect on cell viability on prostate cancer cells. Additionally, the iron uptake and antibody specificity in tumor were consistent with IONPs [92]. Poly (N-isopropylacrylamide-acrylamide-allylamine)-IONPs conjugated with R11 peptide (R11-PIONPs) were developed for specific targeting to prostate cancer. R11-PIONPs were well compatible with normal prostate epithelial cells even the concentration was up to 500 μg/mL. However, R11-PIONPs caused 16% cell death to PC3 and LNCaP cells, besides R11-PIONPs accumulated in PC3 and LNCaP cells in a dose-dependent manner [93]. Fe_3_O_4_ (100 μg/mL) could decrease 15% cell viability of DU145, PC-3, and LNCaP cells. Fe_3_O_4_ (100 μg/mL) could enhance the cytotoxicity of docetaxel (1 nM) in DU145, PC-3, and LNCaP cell lines with 40% cell death [94]. IONPs coated with luteinizing hormone-releasing hormone receptor (LHRH-R) peptide and urokinase-type plasminogen activator receptor (uPAR) peptide (LHRH-AE105-IONPs) were developed as drug delivery system. LHRH-AE105-IONPs were preferential banded and internalized by PC-3 than normal prostate cells. LHRH-AE105-IONPs loaded with paclitaxel (10 ng/mL) remarkably inhibited the PC-3 cell viability for two folds when compared with single-receptor-targeting IONPs [95].

#### Breast carcinoma

MDA-MB-231 cells remarkably enhance the uptake of FeO@HA NPs to fourfold times when compared to the normal cells. In addition, FeO@HA NPs showed well heat generation capability in MRI [96]. Europium-doped IONPs showed no significant cytotoxicity in THP-1, HaCaT, MCF-7 cell lines [97]. Exceedingly small IONPs were synthesized, and showed non-toxicity in human breast cancer cells. The synthetic IONPs exhibited enhanced MRI capability comparable to commercial contrast agents [98]. The cell viability of 4T1 cells were 48.5% after treated with ellipsoidal IONPs (100 μg Fe/mL) under an AMF [99]. The result of cell viability revealed that IONPs@arginine-MTX (Fe-Arg-MTX) could significantly inhibit the survival rate of MCF-7 and 4T1 cell line. The IC_50_ of Fe-Arg-MTX in MCF-7 and 4T1 cell lines were 230 nM and 380 nM for 48 h, respectively [100]. FeO@macrophage membrane (FeO@MM) showed no toxicity in MCF-7 cells even the concentration was up to 800 μg/mL [43]. Dimercaptosuccinic acid modification on the surface of SPIONs could enhance their amount of uptake and prolong the clearance in MCF-7 cells without influencing cell morphology and cell viability, which contributed to targeting breast cancer cells [101]. MXene further modification with tantalum carbide (Ta_4_C_3_) and SPIONs (Ta_4_C_3_-IONP-SPs composite MXenes) were designed as a contrast agent for breast-cancer theranostic. Ta_4_C_3_-IONP-SPs composite MXenes showed excellent biocompatibility in 4T1 cells [102]. Cubic-shaped IONP-poly (amidoamine) dendrimer-Pluronic P123/HSP90α molecular beacon (IPP/MB nanobeacon) was developed for cancer diagnostics and therapy. The IPP/MB nanobeacon (0.5–10 μg Fe/mL) showed good cytocompatibility in MDA-MB-231 and MCF-10A cell lines [103]. Three kind of bioengineered silk named MS1Fe1, MS1Fe2, and MS1Fe1Fe2 were developed to delivery drug to Her2-overexpressing cancer cells. The content of MS1Fe1 silk was positive related to the affinity to IONPs, while negative correlated to the binding to cancer cells. Moreover, the IONPs remarkably enhanced the percentage of apoptosis in SKBR3 cancer cells for 2.5 times [104]. PVPMSFe, as an engineered mesoporous silica-coated FeO, was non-toxic in MCF-7 and HFF2 cells with cell viability more than 80% [105].

#### Cervical cancer

IONPs coated with oleic acid-gelatin shell (Gel-IONPs) could decrease the toxicity and enhance the therapeutic efficacy of Taxol®. The IC_50_ value was 2.28 ± 0.72 ng/mL when the Hela cells were treated with Gel-IONPs for 72 [106]. Polycaprolactone loaded with IONPs could enhance the release of doxorubicin and exert cytotoxic effects on Hela cells under exposure to magnetic hyperthermia [107]^.^ Protein conjugated glutaric acid modified FeO (Pro-Glu-FeO) showed no toxicity in human normal lung cells (WI26VA), but a slight toxicity in HeLa cell line. The cell viability decreased 25% when HeLa cells were exposed to 160 μg/mL of Pro-Glu- FeO for 24 h [108]. USPIO@MIL was biocompatible in RAW 264.7 cells, while showed slight cytotoxicity in Hela cells with 86% viability at a concentration of 20 μg/mL for 24 h [109] (Fig. 5B). The toxicity of three different coating IONPs was assessed in Hela cells. A very low toxicity without morphological alteration was observed after treated with 0.5 mg/mL IONPs for 72 h [110]. SPION modified with heparin-poloxamer (HP) (SPION@HP) were obtained to delivery anticancer drugs, which was highly biocompatible due to the surface coating, and doxorubicin loaded SPION@HP showed significant anticancer effect and low systemic toxicity with 48% Hela cells death at the concentration of 10 μg/mL [111].

#### Gastric carcinoma

Poly (ethylene glycol) (PEG)-coated Fe_3_O_4_ were designed as a miRNA delivery system to enhance the therapeutic effect of Adriamycin (ADR) in gastric cancer cells (SGC7901/ADR cells). The combination of miR16-IONPs with ADR could promote SGC7901/ADR cell apoptosis with slight toxicity (IC_50_ 2.0 mg/mL) [112]. Fe_3_O_4_@Au@β-CD NPs presented excellent biocompatibility to gastric cancer cells, which remained 90% viability at a concentration of 200 μg/mL for 24 h. Additionally, Fe_3_O_4_@Au@β-CD NPs were selectively intake by MGC-803 cells which was observed by confocal laser scanning microscopy. Fe_3_O_4_@Au@β-CD NPs could serve as a potential probe for MRI imaging and targeted drug delivery system [113]. Fe_3_O_4_-carboxymethyl cellulose-5-fluorouracil (Fe_3_O_4_-CMC-5FU, 75 μg/mL) could inhibit the proliferation of SGC7901 cells about 18 ± 0.18% after exposed for 24 h, which was much higher than the pure 5FU. The inhibitory rates at 24, 48, 72 h all indicated that Fe_3_O_4_-CMC-5FU could apparently improve the antitumor effect on SGC7901 cells. The physical and biological mechanism indicated that Fe_3_O_4_-CMC-5FU induced cell death of SGC7901 cells via attacking their mitochondria [114]. Atranorin@SPIONs could obviously inhibit gastric cancer stem cell proliferation when the concentration was up to 12 μg/mL. The inhibition rate of cell proliferation was positively related with the concentration and treatment time of Atranorin@SPIONs [115].

#### Glioma

PEG-neridronate modification improved the biocompatibility of γ-Fe_2_O_3_/CeO_2_ NPs with IC_50_ 2.5 mg/mL, while induced concentration-dependent cytotoxicity in U87MG cells. Additionally, γ-Fe_2_O_3_/CeO_2_@PEG could enter the human glioma cancer cells and induce cell death via autophagy [116]. Zinc@SPIONs were excellent biocompatible in U-87 MG cells at the concentration range 1–100 μg/mL, which could be applied in MRI and magnetic hyperthermia [117]. Ultrasmall SPIONs nanoclusters (SPIOCs)@HSA (paclitaxel)-Arg-Gly-Asp peptides (SPIOCs@HSA(PTX)-RGD) showed no cytotoxicity on U87 cells when the concentration was less than 18 μg/mL [118]. Aurroshell gold@hematite presented minimal toxicity on HUVEC cells, while aurroshell gold@hematite remarkably killed glioblastoma cancer cell when the concentration reached 50 μg/mL. Furthermore, there was a combined effect of aurroshell gold@hematite and hyperthermia, as evidence that 1000 μg/mL of aurroshell gold@hematite could almost kill all U87 cells at 45 ℃ for 1 h [119]. The permeability and uptake of doxorubicin-loaded IONPs significantly enhanced nearly 3 folds in MDCK-MDR1 and U251 cells when compared with pure doxorubicin. Additionally, an external MF has synergetic effect on permeability and cytotoxicity of doxorubicin-loaded IONPs in MDCK-MDR1-glioblastoma model [120].

#### Hepatic carcinoma

γ-FeO-poly (acrylic acid)/poly (serine ester)-*b*-PEG (PICs) presented non-cytotoxicity in MC3T3-E1 and HepG2 cells in the range of concentration (0.751 to 751 μM). The PICs were rapidly degraded to byproducts after exposure for 24 h, and the degradation byproducts were reported to have low cytotoxicity [121]. The cytotoxicity of poly (ethylene glycol) carboxyl-poly (ɛ-caprolactone) modified IONPs (PEG-PCCL-IONPs) were investigated in HepG2 and HEK293 cell lines. The results revealed that PEG-PCCL-IONPs demonstrated little cytotoxicity and induced early apoptosis in HepG2 liver tumor cells at the concentration of 0.25–1.0 mg/mL. However, the viability was negligible affected by PEG-PCCL-IONPs in HEK293 cells [55]. FePd IONPs (20 μg/mL) could remarkably inhibit the production of reactive oxygen species (ROS) and maintain a cell viability more than 90% in HepG2, AGS, SK-MEL-2, MG63, and NCI-H460 cell lines even treated for 7 days [122]. The cell viability, accumulation of ROS, and leakage of transaminase were assessed in primary human hepatocytes and HuH7 tumor cells after treatment with silica coated micrometer-sized IONs (sIONPs) for 5 days. The sIONPs displayed no adverse effects on primary human hepatocytes and HuH7 cells even under the clinical MRI condition [123]. Fe_2_O_3_@bovine serum albumin (Fe_2_O_3_@BSA) were highly compatible in HepG2, 293 T, and rat red blood cell lines when exposed under the concentration of 25–300 μg/mL for 24 h [49]. USPIONs (100 μg/mL Fe) were not cytotoxic in PLC/PRF5 cell after treated with for 48 h at 100 μg/mL Fe [124]. Pullulan stabilized SPIONs (P-SPIONs) were excellent biocompatible, as evidence that the cell viability of HepG2 and l-929 cell lines was more than 90% when exposed to 100 μg/mL P-SPIONs. Interestingly, AMF contributed to the cell death of HepG2 cell when exposed with P-SPIONs [125]. SPIONs@AP613-1 (Apt-USPIO) possessed excellent biocompatibility in Huh-7 and l-02 cells. The cell viability was more than 90% even at concentration of 200 μg/mL Apt-USPIO [68].

#### Osteosarcoma

The suitability of zinc- and cobalt-doped IONPs was assessed in primary human bone marrow-derived mesenchymal stem cells and human osteosarcoma-derived cells. The result showed zinc-doped IONPs possessed strong magnetic property, while cobalt-doped IONPs showed no magnetism. In addition, moderate mixture of both IONPs displayed the optimum magnetic properties without affecting the cytotoxicity [126]. IONPs, functioned with n-hydroxysuccinimide, were conjugated with vascular endothelial growth factor (VEGF) antibody and ligand cluster of differentiation 80 (CD80) (IONPs@CD80 + VEGF) to treat human osteosarcoma. The cell viability was investigated in ATCCTM CRL-2836 cells, which indicated that 1.0 μg/mL of IONPs@CD80 + VEGF could significantly reduce aberrant cell proliferation for 24 h [127]. Hydroxyapatite coated IONPs (IONPs@HA) were cytocompatiable on MG-63 osteosarcoma cells, while pure IONPs showed marked toxicity at the concentration of 120 g/mL for 48 and 72 h. In addition, IONPs@HA could effectively rise the MG-63 cells temperature to 45℃ within 3 min under MF, and almost induce all MG-63 cells death after 30 min exposure [128]. Chitosan (CS)-succinic anhydride (SA)-folic acid (FA) functioned IONPs (IONPs@CS-FA/CS-SA) were non-toxicity in MG-63 cells, while IONPs@CS-FA/CS-SA (20 μM) loaded doxorubicin significantly inhibited cell proliferation with more than 60% cell death for 72 h [129].

#### Lymphoma

FeO coated with hyperbranched polyester (HBPE) with dodecenyl succinic anhydride (DDSA) (FeO/HBPE-DDSA) did not show cytotoxicity in the OCI-LY3 cell even the concentration was up to 100 mg/mL [130]. IONPs remarkably inhibited the growth of diffuse large B-cell lymphoma (DLBCL) cells in a dose-dependent manner via enhancing lipid peroxidation and ferroptosis. The viability of DLBCL cells was less than 30% when treatment with 1200 μg/mL of IONPs [131]. Several rituximab (RTX) antibodies and PEG was conjugated onto the surface of Fe_3_O_4_ to form multivalent nanoprobes. 50 μg Fe/mL of Fe_3_O_4_-PEG-8Ab could decreased the Raji cell viability to 53.8% at 72 h, while the cell viability in same dose Fe_3_O_4_-PEG-2Ab treated sample was 63.7%. Fe_3_O_4_-PEG-nAb showed a valence-dependent manner of Raji cell apoptosis [132]. FeO@MTX and thermo-chemotherapy revealed a synergistic effect on apoptosis in DLBCL line (OCI-LY18) by increasing apoptosis-inducing gene and decreasing apoptosis-inhibiting gene [133]. A large amount of IONPs-quantum dots were phagocytized into A20 mouse B-lymphoma cells, and could accumulated in cells under the influence of external MF. Importantly, IONPs-quantum dots could regulate intracellular non-invasive autophagy and produce proinflammatory cytokine in A20 mouse B-lymphoma cells [134].

#### Renal carcinoma

The cell viability of IONPs@silibinin was tested in A-498 cells, the result indicated that IONPs@silibinin could remarkably inhibit the growth of human kidney cancer cells when compared with pure silibinin (IC_50_ 3 ± 1.76 μg/mL) [135]. SPION conjugated with mAb G250 was designed as an MRI probe to detect renal cell carcinoma, which showed no cytotoxicity in 786–0 renal carcinoma cells at any test concentration (10–100 μg/mL) for 12 h [136].

In general, IONPs can almost inhibit the cell viability of all types of cancer cells, which show great prospects in cancer treatments. The primary reason for the promising anti-cancer effect of IONPs is due to the degradation of iron oxide core, which can induce the excessive ROS production via the Fenton reaction, and then affect the intracellular redox status and iron metabolism [137]. Compared with traditional small molecules, IONPs could release a large amount of iron ions, increase the content of ROS in cells, and thus induce ferroptosis more effectively [138]. Additionally, IONPs regulated the tumor immune microenvironment by affecting apoptosis and autophagy of macrophages, thereby inhibiting tumor development [139, 140]. In addition, the possible application of IONPs for cancer therapy focus on the release and activation of chemotherapy drugs [141], increase of temperature in tumor site under external near-infrared light or magnetic field [142], gene therapy [143], and targeting delivery (including active, passive or magnetic targeting) [144]. However, more biological researches based on the interaction mechanism are required to promote the application of IONPs in cancer therapy.

### IONPs in non-tumor cells

IONPs also have a widely range of applications in non-tumor cells with good cytocompatibility (Table 2). Surface modification and cell types play a vital role in determining iron metabolism in cells. IONPs contribute to osteoblast differentiation and neurite outgrowth.

#### Osteoblast

Nanocomposite scaffolds, containing gelatin (polymer phase), akermanite (ceramic phase), and IONPs were prepared, and showed high photothermal characteristic under NIR laser. Meanwhile, the scaffolds showed low cytotoxicity in G292 osteoblastic cells at the concentration of 0.125–0.50 mg/mL [145]. The influence of biomimetic hybrid scaffolds (Fe-hydroxyapatite/collagen) on MG63 human osteoblast-like cells was investigated, and showed that Fe-hydroxyapatite/collagen significantly promoted the cell proliferation. In addition, Synthesis temperature played the primary role in determining the chemical-physical property of scaffolds. Fe-hydroxyapatite/collagen 25 scaffolds showed the best performance in improving cell proliferation than Fe-hydroxyapatite/collagen 40 and 50 scaffolds [146]. Variable MF mediated by IONPs affected the adipogenic and osteogenic differentiation of human primary adipose derived stem cells. Low intensity of MF exposure within 2 days increased the adipogenesis, while continuous exposure for 7 days contributed to osteogenesis [147].

#### Immune cell

The fluorescent magnetic nanoparticles (α-AP-fmNPs) were designed to manipulate the migration of dendritic cells. α-AP-fmNPs showed no cell toxicity at any concentration (0.3–48 μg/mL), and dramatically improved the migration efficiency of dendritic cells under the influence of magnetic pull force [148]. The influence of SPIONs on dendritic cell migration was investigated by Prussian blue staining and flow cytometry. The result indicated that all dendritic cells were labeled by the SPIONs, and a low dose of SPIONs contributed to the migration of dendritic cells [149]. FeOx NPs (3–22 nm) coated with dopamine sulfonate (DS), zwitterionic, caffeic acid (CAF) and coryneine chloride (COR) were synthesized, respectively. Except for COR-coated FeOx NPs, all FeOx exhibited low internalization and no significantly cytotoxicity in BV2 cells [36]. Three types of macrophage model were used to evaluate the uptake and degradation of IONPs. The result indicated that the coating on the surface of IONPs and macrophage type mainly decided the iron metabolism of IONPs [150].

#### Stem cells

SPIONs-grafted scaffolds could increase 30% length and 62% area of neurite under control of AMF, while the control fiber only increased a 40% length in neurite [151]. Glucosamine-modification could increase cellular uptake and biocompatibility of SPION-poly (acrylic acid) in mesenchymal stem cells without influencing the cell viability [152]. The biocompatibility of gold and IONPs modified with DMSA was investigated in human MSCs. The results indicated that γ-Fe_2_O_3_-DMSA and Au-DMSA could be well uptake and had no significant cytotoxicity in human MSCs [153]. Ruicun, as a SPION agent, did not alter the characteristics of MSCs at the concentration of 200 μg/mL, including cell viability and apoptosis, cell cycle, cell morphology, and osteogenic differentiation [154]. The angiogenic effect of IONPs with curcumin (CMNPs) was investigated in bone marrow-derived MSCs. The result indicated that low concentration (100 to 500 μg/mL) increased the MSCs cell density, while 1000 μg/mL of CMNPs decreased the cell density [155]. Protein-specific molecularly imprinted polymer coating did not influence the biocompatibility or internalization of IONPs in human MSCs, but the imprinted polymer might extend their degradation process from 9 to 21 days [156]. IONPs coated with citric acid (IONPs@CA) just affected cell viability of endothelial cells and MC3T3-E1 cells when the concentration o was up to 100 μg/mL. However, IONPs@CA did not decrease the expression of NO at all concentration [157]. Magnetoferritin could magnetise human MSCs within one minute without changing the characteristics of human MSCs, including membrane integrity, proliferation and multi-lineage differentiation capacity [158]. SPION@silk fibroin could positively regulate the adhesion and proliferation of human bone marrow-derived MSCs, and stimulate osteogenic differentiation when exposed to MF [159]. d-mannose coating could remarkably enhance the internalization of γ-Fe_2_O_3_ in neural stem cells without affecting cell differentiation. However, cell viability was slightly decreased when the dose increased to 0.2 mg/mL [160].

## Clinical applications of IONPs in human

A newly handle probe was designed to detect SPION (Resovist) during surgical process of sentinel lymph node (SLN) with breast cancer. The SLN detection rate in new method was 94.8%, while detection rate in standard radioisotope method was 98.1% [161, 162]. Standard technique which used Technetium-sulphur colloid (99 m Tc) or with blue dye to stage axillary LN had significant radiation. The identification rate of sentinel node biopsy (SNB) based on SPION was 99% in 143 Turkish early breast cancer patients with minimal adverse effect [163]. 12 patients with breast cancer at Uppsala University Hospital were recruited to compare the localization ability of LN with SPION and radioactive tracer. SPION which injected preoperatively 3–15 days could detect all sentinel node, and the axillary signal lasted for 28 days [164]. The detection rate of magnetic technique in 146 early-stage breast cancer patients was 99.3%, which was 0.7% higher than standard technique [165]. Sienna + ® was used as a magnetic tracer for the localization of breast cancer. The identification rate of 108 patients was 97.2% for Sienna + ®, while the standard technique was 95.4% [166]. Magnetic tracer was a safely alternative option for standard technique for SLN mapping and early breast cancer staging, which was low radiation and hypoallergenicity [167–169]. The dosage and time of SPION had been optimized for identifying SLN in melanoma patients. The clinical trial revealed that 1.0 mL SPION with 2 min massage was determined to be the most effective technique [170]. USPIO MRI combined with diffusion-weighted MRI were used to stage bladder and/or prostate cancer patients. The specificity for detection by three readers in 75 patients ranged from 93 to 96% within 9 min, which was much higher than computed tomography [171]. 42 children were treated with 5 mg Fe/kg of ferumoxytol via intravenous injection to stage cancer. Interestingly, the enhancement pattern of ferumoxytol in different type of LN was markedly different, and the accuracy of identification was higher than 90% in children [172].

Cardiovascular magnetic resonance imaging based on ferumoxytol (an USPIO) was compared with conventional gadolinium-based contrast agent in patients with acute myocardial infarction. The results indicated that ferumoxytol provided more detailed pathological features of myocardial infarction with superior safety, which detected tissue-resident macrophage [173]. However, the tissue-resident macrophage was not helpful in detecting myocardial inflammation. Ferumoxytol (4 mg/kg, treated for 3 months) could not display late gadolinium enhancement in patients with acute myocarditis [174]. Ferumoxytol (510 mg Fe) showed potential therapeutic effect in patients with myocardial infarction, as evidenced by decreasing infarct size and enhancing left ventricular function without any adverse effect [175]. The imaging ability of ferumoxytol in macrophage was evaluate in human cerebral aneurysmal. 17 patients were enrolled and assessed the uptake of ferumoxytol in the aneurysms. Results of 2D-gradient-recalled echo indicated that the size of the aneurysms was proportional to the intake of ferumoxytol [176]. 342 patients were enrolled to assess the ability of USPIO to predict aneurysm growth rates and clinical outcomes with abdominal aortic aneurysm. After USPIO-enhanced MRI treatment, 47.3% patients underwent aneurysm rupture or repair, which was 11.7% higher than the control group [177].

28 female patients were recruited to investigate the role of macrophage-mediated inflammation in migraine without aura. USPIO-enhanced 3 T MRI was adopted to detect macrophage-mediated inflammation when migraine-like attack occurred. MRI results showed that macrophage-mediated inflammation was not related to migraine without aura [178]. 18 pediatric patients and 8 healthy adolescents were recruited to evaluate the effect of USPIO-enhanced MRI. Results revealed that 5 mg Fe/kg ferumoxytol could obviously prolong T2* relaxation times to 37.0 ms due to the reduced perfusion and increased edema [179]. Ferumoxytol-enhanced MRI was developed to detect transplanted bone marrow cells in osteonecrosis. Ferumoxytol could prolong the T2* relaxation times of iron-labeled bone marrow cells without influencing bone repair [180]. In summary, IONPs have been applied in clinical practice because of their low radiation and hypoallergenicity. However, ferumoxytol, as a single IONPs commonly used in clinical trials, was mainly used to locate or diagnose breast cancer.

## Conclusions and future outlooks

Because of its unique physical and chemical properties, IONPs have great application potential in biomedical field, including drug targeting, hyperthermia, diagnosis, and cancer treatment. Therefore, there is an urgent need to fully understand the biological effects and toxicity after exposure to IONPs in vitro and in vivo. Although researchers have carried out extensive research on IONPs, there is controversy about their potential toxicity in vivo and in vitro. The main reason is the high variability in the size, surface charge and coatings of IONPs in different studies. In addition, the cell lines, tissues, exposure concentrations and time vary greatly, which affect the biological interaction of IONPs on biological system.

This review aims to fully describe the biological effects and clinic trials of IONPs. Firstly, we summarized the biocompatibility, bio-distribution, metabolism, bio-clearance of IONPs in different animal models. Majority of IONPs were non-toxic and well biocompatible to vital organs of animals, and mainly distributed in the liver and spleen, then quickly cleared by the kidney. Secondly, we described the application of IONPs in different types of tumor cells and non-tumor cells. IONPs selectively targeted to various type of tumor cells and induced tumor cell death without affecting viability and activity of normal cells. The toxicity of IONPs to tumor cells was mainly involved in the shape, surface modification, size, concentration and valence state. Additionally, an applied external magnetic field, radiofrequency generator irradiation, MR imaging and photothermal therapy displayed a synergistic anticancer effect. Meanwhile, IONPs also have a widely range of applications in non-tumor cells with good cytocompatibility. Surface modification and cell types play a vital role in determining iron metabolism in cells. Finally, we reviewed the clinical application of IONPs in the past ten years. Although a variety of IONPs-based nanodrugs have been approved clinically or preclinical trials by the European Medicines Agency (EMA) and United States Food and Drug Administration (FDA) such as NanoTherm® and Feraheme®, Ferumoxytol was still commonly used IONPs in clinic, which was mainly performed to stage or diagnose breast cancer. Toxicity results of IONPs at the cellular level are controversial. Most studies show that IONPs exhibit low toxicity, which is mainly related to size, surface coating, exposure concentration, treatment time, and cell type [181]. Commonly, the cytotoxicity of IONPs is contributed to excess iron ions, ROS production, and oxidative stress. Compared with in vitro studies, in vivo studies can reflect the overall impact of IONPs on the organism more authentically. For example, the size of IONPs can change due to aggregation or forming protein corona with plasma proteins. Up to now, no obvious acute toxicity of IONPs have been reported in vivo, the toxicity of IONPs is mainly manifested in genotoxicity, neurotoxicity, immunotoxicity and reproductive toxicity [182]. Additionally, exposure to IONPs have altered the expression of genes related to oxidative stress, iron transport and apoptosis [183]. The toxicology of IONPs in vivo is predominantly due to size, crystal and dosage of IONPs, as well as age and pathological status of research models. To sum up, it is crucial to establish standard methods for studying the biological effects of IONPs with different physical properties. Additionally, more efforts should be carries out in lab for present and future biomedical applications of IONPs before their clinical or preclinical trials.

## Data Availability

The data that support the findings of this study are available from the authors upon reasonable request.
